# Winning the War against Multi-Drug Resistant Diarrhoeagenic Bacteria

**DOI:** 10.3390/microorganisms7070197

**Published:** 2019-07-10

**Authors:** Chizoba Mercy Enemchukwu, Angus Nnamdi Oli, Ebere Innocent Okoye, Nonye Treasure Ujam, Emmanuel O. Osazuwa, George Ogonna Emechebe, Kenneth Nchekwube Okeke, Christian Chukwuemeka Ifezulike, Obiora Shedrack Ejiofor, Jude Nnaemeka Okoyeh

**Affiliations:** 1 Department of Pharmaceutical Microbiology and Biotechnology, Faculty of Pharmaceutical Sciences, Agulu, Nnamdi Azikiwe University, Awka 420108, Nigeria; 2 Department of Pharmaceutics and Pharmaceutical Technology, Faculty of Pharmaceutical Sciences, Agulu, Nnamdi Azikiwe University, Awka 420108, Nigeria; 3 Department of Pharmaceutical Microbiology and Biotechnology, Faculty of Pharmaceutical Sciences, Enugu State University of Science and Technology, Enugu 400102, Nigeria; 4 Department of Pharmaceutical Microbiology, School of Pharmacy, University of Benin, Benin-City 300271 Edo State, Nigeria; 5 Department of Pediatrics, Chukwuemeka Odumegwu Ojukwu University, Awka 420108, Anambra State, Nigeria; 6 Department of Clinical Laboratory Science, Faculty of Health Sciences, Winston Salem State University, Winston-Salem, NC 27101, USA; 7 Department of Biology and Clinical Laboratory Science, Division of Arts and Sciences, Neumann University, One Neumann Drive, Aston, PA 19014-1298, USA

**Keywords:** Diarrhoeagenic bacteria, bitter leaf, *Vernonia amygdalina*, bitter kola, *Garcinia kola*, *E. coli*, *B. cereus*, *S. aureus*

## Abstract

Drug-resistant-diarrhoeagenic bacteria are currently emerging healthcare challenge. This study investigated the effects of *Vernonia amygdalina*, *Garcinia kola*, tetracycline and metronidazole combinations on such bacteria. Agar well diffusion method was employed to determine the inhibitory effects of the herbal extracts on diarrhoeagenic bacteria while Time-Kill Assay was used to determine bactericidal effects of the extracts against test isolates. Interactions between plant extracts and antibiotics were investigated using Checkerboard assay. Minimum inhibitory concentrations of the extracts against the bacterial isolates ranged between 3.125–50 mg/mL, while those of tetracycline and metronidazole ranged from 30–50 μg/mL. Synergism was observed against *B. cereus* and *S. aureus* for metronidazole + aqueous *G. kola* at all ratios. Generally, the combinations aqueous *G. kola* + ethanolic *G. kola* and aqueous *G. kola* + ethanolic *V. amygdalina* showed more pronounced synergism against the *Staphylococcus aureus* than *B. cereus* isolates with the fractional inhibition concentration (FIC) indices ranging from 0.32–0.95. Synergism of tetracycline + crude extracts and metronidazole combinations were more pronounced on the test isolates and especially on the Gram-negative organisms with FIC indices ranging from 0.41–0.91. Conclusion: The herbal extracts combinations and extracts–antibiotics combinations are synergistic on diarrhoeagenic bacteria at defined combination ratios.

## 1. Introduction

Diarrhea refers to too frequent stooling, daily emissions, or passage of liquid or very soft stool greater than 300 g/day in weight or at least three very loose stools and liquid stools per day and is “the second leading cause of death in children under five years old” [1]. The disease is caused by a wide range of pathogens that find their way into the enteric system and may include mostly bacteria and at times, viruses and parasites. Ingested pathogenic organisms may be confined to the gastrointestinal tract or pathogens may originate from the gut and then spread to other parts of the body where they establish infections or produce toxins injurious to the host cells [2].

The emergence and resurgence of drug resistance have been a big concern in healthcare delivery globally [3,4]. Diarrhoeagenic bacteria are not excluded in the development of resistance to previously effective antibiotics [5,6]. The phenomenal increase in the acquisition of resistance to antimicrobial drugs by pathogens has mostly been attributed to the indiscriminate and improper use of antimicrobials [3,7]. Infections due to *Staphylococcus aureus* are presently resistant to beta-lactams, while *Enterococcus* strains are resistant to vancomycin, ampicillin, gentamycin, and streptomycin [8]. Gram-negative pathogens such as *Salmonella* spp., *Pseudomonas aeruginosa*, and *Klebsiella pneumoniae* have several times been reported to be multi-drug resistant [7,8,9]. The loss of clinical efficacy of such previously effective first-line drugs led to a shift to the second-line or third-line antibiotics which are often more expensive and more troublesome [3,4]. Drug resistance presents an ever-increasing global health threat that involves all major microbial pathogens and antimicrobial drugs [3,4,10]. There have been reported evidences of multi-drug resistant diarrhoeagenic bacteria [5,6,11] around the world including in Nigeria. 

The rural communities of Africa (including Nigeria) and other parts of the world have been using herbal drugs to manage diarrhea caused by bacteria [12,13,14] and the organisms *E. coli*, *B. cereus* and *S. aureus* have all been reported as causing childhood diarrhea [14,15]. An herbal medicine could have similar pharmacological effects as conventional drugs [16] and concomitant use may result in diminution, addition, or potentiation of the two single effects. The implications of herb–drug interactions are, therefore, considered to be multi-dimensional and multi-factorial [16,17].

The plants—*Vernonia amygdalina* and *Garcinia kola*—are well known indigenous plant with several pharmacological activities but it is not yet known, scientifically, if any synergy (and at what combination ratio) exist between them. Also, in the management of multi-drug resistant diarrhoeagenic bacterial infections, we do not have any scientific proof that both plants can be used to control them. This research work, therefore, seeks to evaluate the combined effects of *Vernonia amygdalina* and *Garcinia kola*, locally used individually to manage bacteria diarrhea, as well as the combined effects of the herbal extracts with some orthodox drugs (tetracycline and metronidazole) against diarrhea causing bacteria. This study may justify, or otherwise invalidate, the indigenous use of the plant extracts in the treatment of diarrheal diseases.

## 2. Materials and Methods

### 2.1. Plant Material Source

*Vernonia amygdalina* (bitter leaf) is a household shrub. The leaves were collected from Awka-Etiti while the seeds of *Garcinia kola* (bitter kola) were obtained from local market (Afor-Nnobi) at Nnobi (all in Anambra State of Nigeria). Authentication was by a Taxonomist in the Department of Pharmacognosy and Traditional Medicine, Nnamdi Azikiwe University, Agulu.

### 2.2. Drugs

Pure sample of tetracycline and metronidazole were sourced from Beecham pharmaceuticals, London, United Kingdom.

### 2.3. Test Microorganisms

Diarrhea causing bacteria (two Gram positive—*Bacillus cereus* and *Staphylococcus aureus*—and two Gram negative—*Escherichia coli* and *Salmonella* spp.) were isolated from the stool samples of children (≤5 y) diagnosed of diarrhea at General Hospital Onitsha in Anambra State, after ethical approval of the study protocol was granted by State Ministry of Health (Approval number: MH/PSD:34/VOL.14/525) (Date: 18th January 2016) and parental consent were obtained. The isolates were identified using standard microbiological procedures prior to use. Studies were conducted in the Microbiology Laboratory of the Department of Pharmaceutical Microbiology and Biotechnology, Nnamdi Azikiwe University, Awka, Nigeria.

#### 2.3.1. Preparation of Plant Extracts

Following the collection of the plant materials and drying in the laboratory at 25–30 °C, they were pulverized separately and extracted with water and ethanol. The ethanolic extract was prepared by macerating 400 g of the pulverized sample in 2 L of ethanol and shaken intermittently for 48 h. The mixtures were passed through muslin cloth and filtered further with Whatman filter paper No. 1 (Camlab, Cambridge, UK). The filtrate was concentrated to 50% using rotary evaporator and water bath. The aqueous extraction was done by macerating 400 g of the pulverized sample in 2 L of lukewarm distilled water for 24 h. The mixtures were passed through muslin cloth and the effluxes further filtered with Whatman filter paper No. 1. The filtrates were freeze dried using lyophilizer (York Scientific Industries Pvt. Ltd. New Delhi, India. Name: Yorco Freeze Dryer. Serial number: 08 K033. Model number: YSI-280) and the whole sample stored in the refrigerator for further use.

#### 2.3.2. Antibiotic Susceptibility Testing

The susceptibility testing was performed following the Disc diffusion susceptibility test—a modified Kirby-Bauer method described by Cheesbrough [18]. A sterilized wire loop was used to transfer 3–5 isolated colonies from a Nutrient agar plate into a sterile test tube containing about 4 mL of physiological saline. The colonies were emulsified in the normal saline to obtain a homogeneous suspension of the bacterial cells. The turbidity of the suspension was adjusted visually to that of McFarland 0.5 turbidity standard by adding sterile physiological saline. This served as the inoculum. Using a sterile swab stick, the standardized inoculum in the test tube was then swabbed on the surface of the Mueller–Hinton (MH) agar plate. The antibiotic discs were aseptically placed on the inoculated plates and gently pressed on agar surface using sterilized forceps to ensure proper contact. Plates were inverted within 30 min of applying the discs and incubated aerobically at 35–37 °C for 18–20 h. The inhibition zone diameter (IZD) around each disc was measured in millimeter (mm) using a plastic transparent ruler.

#### 2.3.3. Determination of Antibacterial Activities of Plant Extracts

Stock solutions of the ethanolic and aqueous extracts of the plants were prepared by dissolving 400 mg of the extracts in 2 mL of DMSO respectively (to make 200 mg/mL) in screw capped tubes for primary screening of plant extracts. To investigate the antibacterial activities of plant extracts, the stock solutions were prepared at a concentration of 5000 mg of the extracts in 5 mL of DMSO (to make 1000 mg/mL) in screw capped tubes. Antimicrobial activity of the plant extracts was then assayed using agar well diffusion method as described in previous works [19,20]. Concentrations of 100, 50, 25, 12.5, 6.25, and 3.125 mg/mL were prepared from the stock solutions of the extracts. A 20 mL of molten Mueller–Hinton (MH) agar was aseptically dispensed into sterile Petri dishes (90 mm) and inoculated with 0.1 mL fresh cultures of test isolates at McFarland 0.5 concentration standard and allowed to set. Holes of diameter 6mm were made in the agar plates using a sterile metal cork-borer and to each hole, 60 μL of the various dilutions of each extract and controls were dispensed under aseptic condition. The set up was kept at room temperature in laminar flow machine for 1 h to allow the agents diffuse into the agar medium and then incubated accordingly. Tetracycline (30 μg/mL) and metronidazole (50 μg/mL) were used as positive controls, while sterile water + DMSO were used as negative controls. The plates were then incubated at 37 °C for 24 h; the zones of inhibition were measured and extracts that gave significant activity against test organisms were further tested against test organisms to determine their minimum inhibitory concentrations (MICs).

#### 2.3.4. Determination of Minimum Inhibitory Concentration (MIC) of the Crude Extracts on the Test Isolates

Agar dilution method as described by Akerele et al. [21] was used to determine the MIC of the extracts. The MICs were determined on Mueller Hinton agar using the plant extracts that inhibited the microorganisms. Stock solution (1000 mg/mL) of each extract was prepared by dissolving the extracts in DMSO. The stock solutions were further diluted 2-fold to obtain the following concentrations: 500, 250, 125, 62.5, 31.25, and 15.625 mg/mL. The MICs of the control drugs (tetracycline and metronidazole) were determined using the following concentration of tetracycline—1200, 600, 300, 150, 75, and 37.5 μg/mL and metronidazole—1000, 500, 250, 125, 62.5, and 31.25 μg/mL. Agar plates were prepared by dispensing 9 mL of MH agar into sterile Petri dishes containing 1 mL of the various dilutions of each extract and control drugs. The final plates concentrations were 50, 25, 12.5, 6.25, 3.125, and 1.5625 mg/mL for extracts; 120, 60, 30, 15, 7.5, and 3.75 μg/mL for tetracycline and 100, 50, 25, 12.5, 6.25, and 3.125 μg/mL for metronidazole.

Bacteria cultures grown for 24 h in nutrient broth and standardized to McFarland 0.5 were applied to the surface of the agar plates containing the different concentrations of the extracts and control drugs. The plates were incubated at 37 °C for 18–20 h, after which the plates were observed for growth. The minimum concentrations of the extracts or the drugs that completely inhibit the growth of each microorganism were taken as the MICs. The tests were carried out in triplicate.

#### 2.3.5. Time–Kill Assay of the Extracts

Assays for rate of killing of the bacteria by the crude ethanolic and aqueous extracts were carried out using a modified plating technique as described by Olajuyigbe and Afolayan [22]. Standardized concentrations (McFarland 0.5, equivalent to 1 × 10^8^ CFU/mL) of logarithmic phase culture of test isolates were prepared. The ^1^/_2_ × MIC and 2 × MIC of the extracts were incorporated separately into 10 mL of Mueller Hinton broth in McCartney bottles. A 1 mL volume of the standardized test cultures (1 × 10^8^ CFU/mL) was added to 9 mL of the extract–broth mixture to give a microbial concentration of 1 × 10^7^ CFU/mL. Two controls, one Mueller–Hinton broth without extract at the test concentrations and without the test organisms were included. Next, 0.1 mL of the extract–broth–culture mixture was dispensed in a sterile Petri dish and molten MH agar poured into the plate and allowed to solidify. Samples were taken after 2 h, 4 h, 6 h, and 8 h intervals and analyzed for the effectiveness of extracts against test organisms whether the activity is time or concentration dependent. The procedure was carried out in triplicates. Plates were incubated at 37 °C for 24 h before counting the colonies.

#### 2.3.6. Combined Antimicrobial Activities of the Plant Extracts

Checkerboard method was employed to assay the combined antibacterial activities of the extracts and the standard antibiotics against the test organisms using the agar diffusion method as described by Okore [23]. The extracts were combined with each other and also with the standard antibiotics at concentration of 2 × MIC of individual significant activity against test organisms. The concentrations of the extracts and antibiotics were prepared according to a continuous variation checkerboard technique using the ratio 0:10, 1:9, 2:8, 3:7, 4:6, 5:5, 6:4, 7:3, 8:2, 9:1, and 10:0. The combinations were further serially diluted by a 2-fold process to obtain the final concentrations. Sterile Petri dish containing 20 mL of molten MH agar medium were aseptically inoculated with 0.1 mL fresh cultures of test isolates at McFarland 0.5 concentration standard and allowed to set. Wells (6 mm) were punched in the agar and filled with 60 μL of the various combinations of plant extracts and standard antibiotics. Three replicates of each plate were done. The MICs of each combination was determined.

For each isolate, the fractional inhibitory concentration (FIC) of all combinations were determined and the FIC value for each extract and antibiotic was calculated as

$${\mathrm{FIC}}_{A}=\frac{Conc\;of\;A\;in\;MIC\;A+B}{MIC\;of\;A\;alone}$$

$${\mathrm{FIC}}_{B}=\frac{Conc\;of\;B\;in\;MIC\;A+B}{MIC\;of\;B\;alone}$$

According to Okore [23], FIC index <1.0 means Synergism, = 1 means additivity, >1 but less than 2 means indifference while ≥2 means antagonism.

## 3. Results

Table 1 shows the yields after extraction and lyophilization for the two plants. The ethanolic extracts present a better extraction yield.

**Figure 1:**
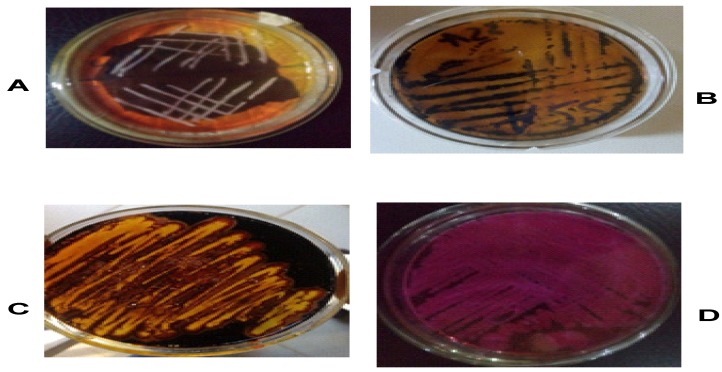
Identification of Test Isolates on Selective Media. (**A**) *Staphylococcus aureus* in Mannitol Salt Agar, (**B**) *Salmonella* spp. in *Salmonella-Shigellae* Agar, (**C**) *Bacillus cereus* in Starch Agar and (**D**) *Escherichia coli* in MacConkey Agar.

The result of the confirmatory tests for the diarrhea causing bacteria isolates (*Bacillus cereus*, *Escherichia coli*, *Staphylococcus aureus*, and *Salmonella* spp.) are shown in Figure 1. Mannitol salt agar identified the presence of *Staphylococcus aureus* (creamy big cluster colonies); *Salmonella-shigellae* agar was used to identify the presence of *Salmonella* spp. (blackish rod like colonies), starch agar revealed the presence of *Bacillus cereus* (yellow colored slightly opalescent gel—clearing around the colony) while MacConkey agar helped in the identification of *E. coli* (dry pinkish colonies) as previously reported by Cheesbrough [18].

The antibiotic susceptibility profile of the test organisms is shown in Table 2. The microorganisms were found to be resistant to many of the standard antibiotics used.

Multi-Antibiotic Resistance Index (MARI) Calculations: The Multi-Antibiotic Resistance Indices of the isolates were calculated as *x/y* [24,25].

Where *x* is the number of antibiotics to which the isolate was resistant and *y* is the number of antibiotics to which the isolate was subjected = 11.

All the isolates have a MARI of more than 20% with three-quarter of them having a MARI of even greater than 50% (Table 3). Higher MARI signifies high multi-drug resistance character.

The antibacterial activities of the aqueous and ethanolic extracts of *Garcinia kola* on the test isolates are shown in Table 4 indicating that the isolates were more susceptible to the ethanolic extracts of the tested plants compared to the aqueous extracts. The zones of inhibition exhibited by the extracts against the tested bacterial isolates ranged between 0.0 to 9.0 mm.

The antibacterial activities of the aqueous and ethanolic extracts of *Vernonia amygdalina* on the test isolates are shown in Table 5. Isolates showed higher susceptibility to the ethanolic extract compared to the aqueous extracts. The zones of inhibition exhibited by the extracts against the tested bacterial isolates ranged between 0.0 to 15.0 mm.

The crude ethanolic extracts of the plants were more active against the test isolates (with MIC range 3.125–6.25 mg/mL) than the crude aqueous extracts of the plants (with MIC range 12.5–50 mg/mL) Table 6.

The FIC indices for combination of crude extracts and then combination of the extracts with either metronidazole or tetracycline ranged from 6.55–0.21 as shown in Table 7, Table 8, Table 9 and Table 10. The combination of metronidazole and tetracycline showed mostly synergism against all test isolates. The combination of ethanolic *V. amygdalina* extract and ethanolic *G. kola* were synergistic only against *S. aureus* and *E. coli* and at selected combinations.

The tetracycline/ethanolic *V. amygdalina* extract combinations showed synergism mostly against *E. coli* compared to the other isolates and least synergy against *B. cereus*. Tetracycline/ethanolic *G. kola* extract combination performed better against the test isolates than the tetracycline/ethanolic *V. amygdalina* combination.

Metronidazole/ethanolic *V. amygdalina* extract combination was observed to show synergy only against *E. coli* and at selected combination ratios. However, the results were better than combinations of metronidazole with ethanolic *G. kola* extract. Metronidazole/aqueous *G. kola* extract combinations performed better than the metronidazole/ethanolic *G. kola* extract combination. For *B. cereus* and *S. aureus* diarrhoea, the best treatment appears to be metronidazole/aqueous *G. kola* extract combinations.

When the extracts were combined, the E.*G*. /A.*G*. and E.*V*./A.*G* combinations gave excellent results against *S. aureus*. The E.*V*. /A.*G*. combination proved to be a wrong combination.

Generally, the combinations aqueous *G. kola* + ethanolic *G. kola* and aqueous *G. kola* + ethanolic *V. amygdalina* showed more pronounced synergistic effects against the *Staphylococcus aureus* than *B. cereus* isolates with the fractional inhibition concentration (FIC) indices ranging from 0.32–0.95. The synergistic effects of tetracycline + crude extracts and metronidazole combinations were more pronounced on the test isolates and especially on the Gram-negative organism (*E. coli*) than *B. cereus* and *S. aureus* with FIC indices ranging from 0.41–0.91.

**Figure 2:**
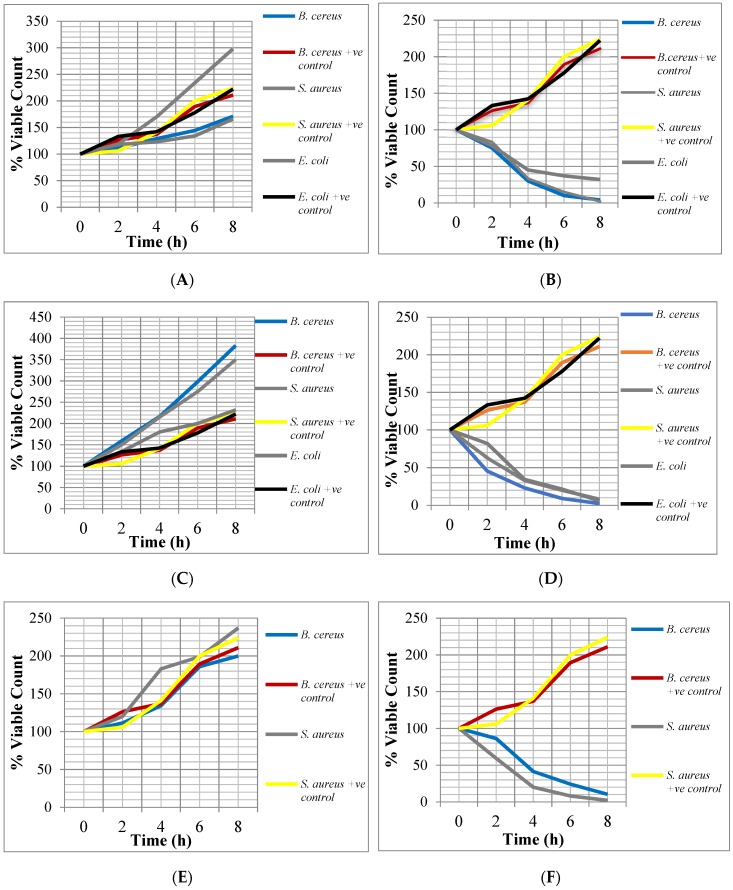
Bactericidal activity of the crude extracts against the test isolates. +ve control = without extracts. (**A**) ½ × minimum inhibitory concentration (MIC) of ethanolic extract of *G. kola*; (**B**) 2 × MIC of ethanolic extract of *G. kola*; (**C**) ½ × MIC of ethanolic extract of *V. amygdalina*; (**D**) 2 × MIC of ethanolic extract of *V. amygdalina*; (**E**) ½ × MIC of aqueous extract of *G. kola*; (**F**) 2 × MIC of aqueous extract of *G. kola*.

From Figure 2, it can be observed that the crude extracts of the two plants showed bactericidal activity against *E. coli*, *S. aureus* and *B. cereus* during the Time-Kill assay. After 2–4 h of incubating the bacteria with the ½ x MICs and 2 x MICs, the percentage viable cell count ranged between 216.2% and 20%. After 6–8 h of incubation with these different concentrations, the percentage viable cell count ranged between 186% and 2%. The bactericidal activity of the crude extracts against the test isolates increased with twice the MICs of the extracts but decreased at half their MICs.

## 4. Discussion

Indiscriminate use of antibiotics among the general population has favoured the emergence of resistant strains. Multi-drug resistance was observed in the test bacterial isolates with a MARI > 20%. Similar results were shown in earlier studies and results were attributed to selective pressure due antibiotic misuse [7,25,26]. The susceptibility testing results are in line with the findings of antibiotic susceptibility tests done by other researchers [27,28] which showed that the isolates (*Escherichia coli*, *Bacillus cereus*, *Staphylococcus aureus*, and *Salmonella* spp.) are susceptible to some of the antibiotics used in this work (ofloxacin, ciprofloxacin, gentamicin, erythromycin, and ceftriaxone). The isolate *Bacillus cereus* is particular resistant to the commercial antibiotics tested

Generally, the ethanolic extracts were more active than the aqueous extracts. They showed broader spectrum activity against the test isolates while the aqueous extracts showed less spectrum of activity. This could be attributed to the poor extractive power/strength of water as a solvent.

The ethanolic extracts of *Vernonia amygdalina* leaves showed greater activity against *S. aureus, B. cereus* and *E. coli*. The inactivity or low antibacterial activity of the aqueous *V. amygdalina* extract confirmed the work of Sule and Agbabiaka [29] who recorded no antimicrobial activity of its aqueous extracts on strains of *E. coli, Klebsiella* spp., and *Shigellae* spp. and also reduced activity on *Salmonella* spp. compared with the ethanolic extracts of the plant. The ethanolic extract of *G. kola* seed showed activity against all the test isolates except *Salmonella* while the aqueous extract showed activity against *B. cereus* and *S. aureus*. This may be due to the fact that ethanol was found to be a better solvent of extraction of the active principles of medicinal importance in plant [30].

Ethanolic crude extract of *V. amygdalina* displayed lower MICs (i.e., higher inhibitory activity) against the Gram-positive isolates than the Gram-negative isolates while aqueous *G. kola* recorded high MICs (i.e. lower inhibitory activity) on test isolate (*Staphylococcus aureus*). The difference between their MICs against the test isolate was extremely statistically significant (*p* < 0.0007) compared with the statistical significances of ethanolic extracts of *V. amygdalina* and *G. kola* combined with the antibiotics (E.*V.* vs. Metronidazole = 0.001, E.*G.* vs. Metronidazole = 0.01, E.*V.* vs. Tetracycline = *p* < 0.05). This is consistent with the report by Ghamba et al. [20], on in vitro antibacterial activity of crude ethanol, acetone, and aqueous *G. kola* seed extracts on selected clinical isolates. Also, Adetunji et al. [31] investigated the bacterial activity of crude extracts of *V. amygdalina* on clinical isolates and reported that cold and hot ethanolic extract of *V. amygdalina* rather than the aqueous extract of *V. amygdalina* produced effective antimicrobial activities against *S. aureus*, *P. aeruginosa* and *E. coli*; and the MICs were 25, 50, and 50 mg/mL for *S. aureus*, *P. aeruginosa* and *E. coli* respectively, while the aqueous extract of *V. amygdalina* did not show activity.

The ethanol extracts showed more activity against the bacteria isolates than the water extracts. This may be due to the higher polarity of the ethanol which tends to extract more active compounds from the plants than water [31]. The killing ability of the plant extracts are quantity and time dependent. The growing problem of multi-drug-resistant pathogens has posed an age-long concern in the choice of antimicrobial therapy [32]. Synergism from the combination of antimicrobial agents with crude plant extracts was verified and the results revealed the importance of plant extracts when combined with antibiotics to control diarrhoeagenic bacteria. The combinations (aqueous *G. kola* + ethanolic *G. kola* and aqueous *G. kola* + ethanolic *V. amygdalina*) showed more pronounced synergistic effects against *Staphylococcus aureus* than *Bacillus cereus*. The synergistic effects of tetracycline + crude extracts (ethanolic *G. kola,* aqueous *G. kola* and ethanolic *V. amygdalina*) and metronidazole + crude extracts combinations were pronounced on the test isolates. The improvement in the activity of tetracycline and the crude extract may be due to the accumulation of inhibitory concentrations at the target site or due to other mechanisms. The combinations of metronidazole and crude ethanolic and aqueous extracts *G. kola* and *V. amygdalina* showed more indifference and less antagonistic effects. However, the combination of metronidazole with aqueous *G. kola* recorded synergistic activities against *B. cereus and S. aureus.* According to Chimezie et al. [33], synergistic activity suggests different mode of actions of the combining compounds. Combined antimicrobials are preferred as microbial resistance is less likely to develop against substances having more than one mode of action [34]. The antibacterial activities of all the extracts either when used separately or combined were concentration dependent as zones of growth inhibition increased with increasing concentration of the extracts. Some researchers had reported that the efficacy of most plant extracts is concentration-dependent [35,36].

## 5. Conclusions

Drug resistant diarrhoeagenic bacterial infections can be controlled through a careful combination of *Vernonia amygdalina* and *Garcinia kola* leaves extracts or their combinations with tetracycline or metronidazole in the right (synergistic) ratio. The detection of synergy between the crude extracts demonstrates the potential of these plants as sources of antibiotic-resistance-modifying compounds for the control of diarrhoeagenic bacterial infections. Finally, the results of this study give some scientific credence to the indigenous use of the plant extracts for the treatment of diarrheal diseases.

## Figures and Tables

**Table 1:** Percentage yield of crude extract obtained from each plant.

Plants	Percentage Yield (%)	
	Aqueous Extracts	Ethanol Extracts
***Garcinia kola* (bitter kola)**	2.6	7.5
***Vernonia amygdalina***	3.2	8.4

**Table 2:** The Mean inhibition zone diameter (IZD) (in mm) produced by various antibiotics against the test organism.

Antibiotics	Inhibition Zone Diameter (mm)			
	*Bacillus cereus*	*Staphylococcus aureus*	*Escherichia coli*	*Salmonella* spp.
**Ceftaxidime (30 μg)**	0 ± 0	0 ± 0	0 ±0	0 ± 0
**Cefuroxime (30 μg)**	0 ± 0	0 ± 0	0 ± 0	0 ± 0
**Gentamicin (10 μg)**	14 ± 4.67	26 ± 8.67	17 ± 5.67	16 ± 5.33
**Cefixime (5 μg)**	0 ± 0	0 ± 0	0 ± 0	0 ± 0
**Ofloxacin (5 μg)**	20 ± 6.67	27 ± 9	23 ± 7.67	29 ± 9.67
**Augmentin (30 μg)**	0 ± 0	18 ± 6	5 ±1.67	0 ± 0
**Nitrofurantoin (5 μg)**	0 ± 0	30 ± 10	20 ± 6.67	23 ± 7.67
**Ciprofloxacin (5 μg)**	15 ± 5	32 ± 10.67	23 ± 7.67	23 ± 7.67
**Ceftriaxone (30 μg)**	16 ± 5.33	14 ± 4.67	14 ± 4.67	26 ± 8.67
**Erythromycin (5 μg)**	0 ± 0	26 ± 8.67	8 ± 2.67	0 ± 0
**Cloxacillin (5 μg)**	0 ± 0	20 ± 6.67	0 ± 0	0 ± 0

X: mean; SEM: standard error of mean (X ± SEM).

**Table 3:** Multiple Antibiotic Resistance Index.

Isolates	*x*	Multiple Antibiotic Resistance Index (MARI)	MARI in %	Remark
***Bacillus cereus***	8	0.7273	72.73	>20%
***Staphylococcus aureus***	4	0.3636	36.36	>20%
***Escherichia coli***	7	0.6364	63.64	>20%
***Salmonella* spp.**	6	0.5455	54.55	>20%

**Table 4:** Antibacterial activity of the crude extracts of *Garcinia kola* against test isolates.

Concentration of Extracts (mg/mL)		IZDs of Extracts (mm)			
		*Bacillus cereus*	*Escherichia coli*	*Staphylococcus aureus*	*Salmonella* spp.
**Ethanolic extract**	100.00	9 ± 0.33	7 ± 0.33	9 ± 0.88	0 ± 0
	50.00	8 ± 1.00	6 ± 0.88	7 ± 0.58	0 ± 0
	25.00	7 ± 0.33	5 ± 1.00	5 ± 0.67	0 ± 0
	12.50	6 ± 0.33	2 ± 0.58	3 ± 1.45	0 ± 0
	6.25	5 ± 0.67	0 ± 0	0 ± 0	0 ± 0
	3.125	4 ± 0.67	0 ± 0	0 ± 0	0 ± 0
**Aqueous extract**	100.00	6 ± 0.88	0 ± 0	9 ± 0.58	0 ± 0
	50.00	6 ± 0.33	0 ± 0	6 ± 0.33	0 ± 0
	25.00	6 ± 0.33	0 ± 0	4 ± 1.15	0 ± 0
	12.50	5 ± 0.33	0 ± 0	2 ± 0.33	0 ± 0
	6.25	2 ± 0.88	0 ± 0	0 ± 0	0 ± 0
	3.125	0 ± 0	0 ± 0	0 ± 0	0 ± 0
**Positive control**	Tetracycline (30 µg)	5	11	9	0
	Metronidazole (50 µg)	5	3	4	0
**Negative control**	DMSO + Water	0	0	0	0

X: mean; SEM: standard error of mean (X ± SEM).

**Table 5:** Antibacterial activity of the crude extracts of *Vernonia amygdalina* against test isolates.

Concentration of Extracts (mg/mL)		IZDs of Extracts (mm)			
		*Bacillus cereus*	*Escherichia coli*	*Staphylococcus aureus*	*Salmonella* spp.
**Ethanolic Extract**	100.00	14 ± 0.33	15 ± 0.58	14 ± 0.33	0 ± 0
	50.00	11 ± 0.67	10 ± 0.88	11 ± 0.58	0 ± 0
	25.00	8 ± 0.58	6 ± 0.88	7 ± 0.67	0 ± 0
	12.50	5 ± 0.33	3 ± 0.58	5 ± 0.33	0 ± 0
	6.25	3 ± 0.58	0 ± 0	5 ± 0.58	0 ± 0
	3.125	0 ± 0	0 ± 0	0 ± 0	0 ± 0
**Aqueous Extract**	100.00	0 ± 0	0 ± 0	0 ± 0	0 ± 0
	50.00	0 ± 0	0 ± 0	0 ± 0	0 ± 0
	25.00	0 ± 0	0 ± 0	0 ± 0	0 ± 0
	12.50	0 ± 0	0 ± 0	0 ± 0	0 ± 0
	6.25	0 ± 0	0 ± 0	0 ± 0	0 ± 0
	3.125	0 ± 0	0 ± 0	0 ± 0	0 ± 0
**Positive control**	Tetracycline (30 µg)	5	11	9	0
	Metronidazole (50 µg)	5	3	4	0
**Negative control**	DMSO + Water	0	0	0	0

X: mean; SEM: standard error of mean (X ± SEM).

**Table 6:** MICs of crude extracts of *V. amygdalina* and *G. kola* and control drugs against test isolates.

Test Isolates	Minimum Inhibitory Concentrations					
	Ethanolic *Garcinia kola* (mg/mL)	Ethanolic *Vernonia amygdalina* (mg/mL)	Aqueous *Garcinia kola* (mg/mL)	Control (Tetracycline μg/mL)	Control (Metronidazole μg/mL)	DMSO Negative Control
***Bacillus cereus***	6.25	3.125	12.5	30	50	0
***Escherichia coli***	6.25	6.25	-	30	50	0
***S. aureus***	6.25	3.125	50	30	50	0

**Table 7:** Effect of the extract and antibiotic combinations on test isolates.

***B. cereus***					
**T:M**	**∑FIC**	**Interpretation of Result**	**E.*V.*:E.*G.***	**∑FIC**	**Interpretation of Result**
**10:0**	-	-	**10:0**	-	**-**
**9:1**	0.92	Synergism	**9:1**	1.20	Indifference
**8:2**	0.84	Synergism	**8:2**	1.05	Indifference
**7:3**	1.62	Indifference	**7:3**	2.09	Antagonism
**6:4**	0.73	Synergism	**6:4**	2.08	Antagonism
**5:5**	0.62	Synergism	**5:5**	1.04	Indifference
**4:6**	0.57	Synergism	**4:6**	2.05	Antagonism
**3:7**	0.50	Synergism	**3:7**	1.01	Indifference
**2:8**	0.82	Synergism	**2:8**	2.04	Antagonism
**1:9**	0.66	Synergism	**1:9**	2.00	Antagonism
**0:10**	-	-	**0:10**	-	**-**
***S. aureus***					
**T:M**	**∑FIC**	**Interpretation of Result**	**E.*V.*:E.*G.***	**∑FIC**	**Interpretation of Result**
**10:0**	-	-	**10:0**	-	**-**
**9:1**	0.90	Synergism	**9:1**	1.90	Indifference
**8:2**	0.82	Synergism	**8:2**	0.46	Synergism
**7:3**	0.78	Synergism	**7:3**	1.84	Indifference
**6:4**	0.68	Synergism	**6:4**	1.84	Indifference
**5:5**	0.55	Synergism	**5:5**	0.76	Synergism
**4:6**	0.50	Synergism	**4:6**	2.83	Antagonism
**3:7**	0.50	Synergism	**3:7**	0.39	Synergism
**2:8**	0.31	Synergism	**2:8**	0.61	Synergism
**1:9**	0.22	Synergism	**1:9**	0.55	Synergism
**0:10**	-	-	**0:10**	-	**-**
***E. coli***					
**T:M**	**∑FIC**	**Interpretation of Result**	**E.*V.*:E.*G.***	**∑FIC**	**Interpretation of Result**
**10:0**	-	-	**10:0**	-	
**9:1**	0.90	Synergism	**9:1**	1.11	Indifference
**8:2**	0.82	Synergism	**8:2**	0.63	Synergism
**7:3**	0.78	Synergism	**7:3**	1.30	Indifference
**6:4**	0.68	Synergism	**6:4**	0.70	Synergism
**5:5**	0.55	Synergism	**5:5**	1.50	Indifference
**4:6**	0.50	Synergism	**4:6**	0.80	Synergism
**3:7**	0.41	Synergism	**3:7**	0.85	Synergism
**2:8**	0.31	Synergism	**2:8**	0.90	Synergism
**1:9**	0.22	Synergism	**1:9**	0.48	Synergism
**0:10**	-	-	**0:10**	-	

T = Tetracycline; M = Metronidazole; E. *V. =* Ethanolic *V. amygdalina* extract; E. *G.* = Ethanolic *G. kola* extract while A. *G.* = Aqueous *G. kola* extract, FIC: fractional inhibition concentration.

**Table 8:** Effect of tetracycline and crude extracts combinations on test isolates.

***B. cereus***								
**T:E.*V***	**∑FIC**	**Interpretation of Result**	**T:E.*G***	**∑FIC**	**Interpretation of Result**	**T:A.*G***	**∑FIC**	**Interpretation of Result**
**10:0**	-	-	**10:0**	-	-	**10:0**	-	-
**9:1**	1.00	Additive	**9:1**	0.95	Synergism	**9:1**	1.00	Additivity
**8:2**	1.80	Indifference	**8:2**	0.90	Synergism	**8:2**	0.90	Synergism
**7:3**	3.40	Antagonism	**7:3**	0.88	Synergism	**7:3**	0.89	Synergism
**6:4**	1.60	Indifference	**6:4**	1.62	Indifference	**6:4**	0.83	Synergism
**5:5**	1.49	Indifference	**5:5**	0.75	Synergism	**5:5**	0.80	Synergism
**4:6**	0.73	Synergism	**4:6**	0.73	Synergism	**4:6**	0.72	Synergism
**3:7**	1.35	Indifference	**3:7**	0.67	Synergism	**3:7**	1.33	Indifference
**2:8**	1.24	Indifference	**2:8**	0.62	Synergism	**2:8**	2.40	Antagonism
**1:9**	1.13	Indifference	**1:9**	1.11	Indifference	**1:9**	2.22	Antagonism
**0:10**	-	-	**0:10**	-	-	**0:10**	-	-
***S. aureus***								
**T:E.*V***	**∑FIC**	**Interpretation of Result**	**T:E.*G***	**∑FIC**	**Interpretation of Result**	**T:A.*G***	**∑FIC**	**Interpretation of Result**
**10:0**	-	-	**10:0**	-	-	**10:0**	-	-
**9:1**	1.90	Indifference	**9:1**	0.92	Synergism	**9:1**	1.00	Additivity
**8:2**	1.80	Indifference	**8:2**	0.85	Synergism	**8:2**	1.00	Additivity
**7:3**	1.73	Indifference	**7:3**	0.82	Synergism	**7:3**	1.02	Indifference
**6:4**	0.84	Synergism	**6:4**	0.73	Synergism	**6:4**	1.02	Indifference
**5:5**	0.75	Synergism	**5:5**	0.62	Synergism	**5:5**	1.00	Additivity
**4:6**	0.73	Synergism	**4:6**	0.57	Synergism	**4:6**	1.03	Indifference
**3:7**	0.68	Synergism	**3:7**	0.49	Synergism	**3:7**	1.02	Indifference
**2:8**	0.62	Synergism	**2:8**	0.42	Synergism	**2:8**	1.00	Additivity
**1:9**	0.57	Synergism	**1:9**	1.32	Synergism	**1:9**	1.01	Indifference
**0:10**	-	-	**0:10**	-	-	**0:10**	-	-
***E. coli***								
**T:E.*V***	**∑FIC**	**Interpretation of Result**	**T:E.*G***	**∑FIC**	**Interpretation of Result**	**T:A.*G***	**∑FIC**	**Interpretation of Result**
**10:0**	-	-	**10:0**	-	-	**10:0**	-	-
**9:1**	0.91	Synergism	**9:1**	0.91	Synergism	**9:1**	-	-
**8:2**	0.83	Synergism	**8:2**	0.82	Synergism	**8:2**	-	-
**7:3**	0.78	Synergism	**7:3**	0.78	Synergism	**7:3**	-	-
**6:4**	0.68	Synergism	**6:4**	0.68	Synergism	**6:4**	-	-
**5:5**	0.56	Synergism	**5:5**	0.55	Synergism	**5:5**	-	-
**4:6**	0.50	Synergism	**4:6**	0.50	Synergism	**4:6**	-	-
**3:7**	0.41	Synergism	**3:7**	0.41	Synergism	**3:7**	-	-
**2:8**	0.62	Synergism	**2:8**	0.62	Synergism	**2:8**	-	-
**1:9**	0.44	Synergism	**1:9**	0.43	Synergism	**1:9**	-	-
**0:10**	-	-	**0:10**	-	-	**0:10**	-	-

T = Tetracycline; M = Metronidazole; E. *V. =* Ethanolic *V. amygdalina* extract; E. *G.* = Ethanolic *G. kola* extract while A. *G.* = Aqueous *G. kola* extract.

**Table 9:** Effect of metronidazole and crude extracts combinations on test isolates.

***B. cereus***								
**M:E.*V***	**∑FIC**	**Interpretation of Result**	**M:E.*G***	**∑FIC**	**Interpretation of Result**	**M:A.*G***	**∑FIC**	**Interpretation of Result**
**10:0**	-	-	**10:0**	-	-	**10:0**	-	-
**9:1**	1.10	Indifference	**9:1**	1.30	Indifference	**9:1**	0.32	Synergism
**8:2**	2.42	Antagonism	**8:2**	1.60	Indifference	**8:2**	0.30	Synergism
**7:3**	5.20	Antagonism	**7:3**	1.91	Indifference	**7:3**	0.33	Synergism
**6:4**	2.80	Antagonism	**6:4**	1.10	Indifference	**6:4**	0.35	Synergism
**5:5**	1.50	Indifference	**5:5**	0.63	Synergism	**5:5**	0.43	Synergism
**4:6**	3.20	Antagonism	**4:6**	0.72	Synergism	**4:6**	0.80	Synergism
**3:7**	3.40	Antagonism	**3:7**	1.61	Indifference	**3:7**	0.50	Synergism
**2:8**	1.80	Indifference	**2:8**	3.40	Antagonism	**2:8**	0.50	Synergism
**1:9**	1.90	Indifference	**1:9**	1.84	Indifference	**1:9**	0.48	Synergism
**0:10**	-	-	**0:10**	-	-	**0:10**	-	-
***S. aureus***								
**M:E.*V***	**∑FIC**	**Interpretation of Result**	**M:E.*G***	**∑FIC**	**Interpretation of Result**	**M:A.*G***	**∑FIC**	**Interpretation of Result**
**10:0**	-	-	**10:0**	-	-	**10:0**	-	-
**9:1**	1.70	Indifference	**9:1**	1.30	Indifference	**9:1**	0.21	Synergism
**8:2**	1.62	Indifference	**8:2**	1.60	Indifference	**8:2**	0.30	Synergism
**7:3**	3.10	Antagonism	**7:3**	1.30	Indifference	**7:3**	0.42	Synergism
**6:4**	3.80	Antagonism	**6:4**	1.40	Indifference	**6:4**	0.52	Synergism
**5:5**	1.25	Indifference	**5:5**	1.50	Indifference	**5:5**	0.61	Synergism
**4:6**	2.80	Antagonism	**4:6**	1.61	Indifference	**4:6**	0.71	Synergism
**3:7**	3.10	Antagonism	**3:7**	0.21	Synergism	**3:7**	0.74	Synergism
**2:8**	3.50	Antagonism	**2:8**	0.90	Synergism	**2:8**	0.83	Synergism
**1:9**	1.90	Indifference	**1:9**	1.91	Indifference	**1:9**	0.91	Synergism
**0:10**	-	-	**0:10**	-	-	**0:10**	-	-
***E. coli***								
**M:E.*V***	**∑FIC**	**Interpretation of Result**	**M:E.*G***	**∑FIC**	**Interpretation of Result**	**M:A.*G***	**∑FIC**	**Interpretation of Result**
**10:0**	-	-	**10:0**	-	-	**10:0**	-	-
**9:1**	1.91	Indifference	**9:1**	2.20	Antagonism	**9:1**	-	-
**8:2**	1.80	Indifference	**8:2**	2.40	Antagonism	**8:2**	-	-
**7:3**	1.70	Indifference	**7:3**	1.30	Indifference	**7:3**	-	-
**6:4**	0.80	Synergism	**6:4**	1.40	Indifference	**6:4**	-	-
**5:5**	0.40	Synergism	**5:5**	0.75	Synergism	**5:5**	-	-
**4:6**	0.70	Synergism	**4:6**	1.61	Indifference	**4:6**	-	-
**3:7**	0.70	Synergism	**3:7**	0.90	Synergism	**3:7**	-	-
**2:8**	0.60	Synergism	**2:8**	1.80	Indifference	**2:8**	-	-
**1:9**	1.10	Indifference	**1:9**	1.91	Indifference	**1:9**	-	-
**0:10**	-	-	**0:10**	-	-	**0:10**	-	-

T = Tetracycline; M = Metronidazole; E.*V. =* Ethanolic *V. amygdalina* extract; E.*G.* = Ethanolic *G. kola* extract while A.*G.* = Aqueous *G. kola* extract.

**Table 10:** Effect of crude extracts (aqueous and ethanolic) combinations on test isolates.

***B. cereus***					
**E.*G*:A.*G***	**∑FIC**	**Interpretation of Result**	**E.*V.*:A.*G.***	**∑FIC**	**Interpretation of Result**
**10:0**	-	-	**10:0**	-	-
**9:1**	0.95	Synergism	**9:1**	3.90	Antagonisms
**8:2**	0.92	Synergism	**8:2**	1.90	Indifference
**7:3**	0.85	Synergism	**7:3**	3.76	Antagonisms
**6:4**	1.61	Indifference	**6:4**	6.55	Antagonisms
**5:5**	0.80	Synergism	**5:5**	6.10	Antagonisms
**4:6**	0.71	Synergism	**4:6**	1.44	Indifference
**3:7**	1.32	Indifference	**3:7**	5.31	Antagonisms
**2:8**	1.21	Indifference	**2:8**	4.90	Antagonisms
**1:9**	2.21	Antagonism	**1:9**	4.45	Antagonisms
**0:10**	-	-	**0:10**	-	-
***S. aureus***					
**E.*G.*:A.*G.***	**∑FIC**	**Interpretation of Result**	**E.*V.*:A.*G.***	**∑FIC**	**Interpretation of Result**
**10:0**	-	-	**10:0**	-	-
**9:1**	0.32	Synergism	**9:1**	0.61	Synergism
**8:2**	0.41	Synergism	**8:2**	0.61	Synergism
**7:3**	0.49	Synergism	**7:3**	0.72	Synergism
**6:4**	0.57	Synergism	**6:4**	0.73	Synergism
**5:5**	0.64	Synergism	**5:5**	0.77	Synergism
**4:6**	0.71	Synergism	**4:6**	0.82	Synergism
**3:7**	0.78	Synergism	**3:7**	0.85	Synergism
**2:8**	0.85	Synergism	**2:8**	0.90	Synergism
**1:9**	0.93	Synergism	**1:9**	0.95	Synergism
**0:10**	-	-	**0:10**	-	-

T = Tetracycline; M = Metronidazole; E. *V. =* Ethanolic *V. amygdalina* extract; E. *G.* = Ethanolic *G. kola* extract while A. *G.* = Aqueous *G. kola* extract.
